# Group A Streptococcal Puerperal Sepsis: Historical Review and 1990s Resurgence

**DOI:** 10.1155/S1064744994000190

**Published:** 1994

**Authors:** Lawrence Nathan, Kenneth J. Leveno

**Affiliations:** 1 Department of Obstetrics and Gynecology University of Texas Southwestern Medical School Dallas TX USA; 2 Department of Gynecology and Obstetrics Emory University School of Medicine 69 Butler Street, S.E. Atlanta GA 30303 USA

## Abstract

There appears to be a resurgence of puerperal sepsis due to a historically important pathogen, group A β-hemolytic streptococcus.


Infectious Diseases in Obstetrics and Gynecology 1:252–255 (1994)
© 1994 Wiley-Liss, Inc.

### Group A Streptococcal Puerperal Sepsis: Historical Review and 1990s Resurgence

**Lawrence Nathan and Kenneth J. Leveno**
*Department of Obstetrics and Gynecology, University of Texas Southwestern Medical School, Dallas, TX*

ABSTRACT

**There appears to be a resurgence of puerperal sepsis due to a historically important pathogen, group A β-hemolytic streptococcus.** © 1994 Wiley-Liss, Inc.

KEY WORDS
Group A streptococcus, puerperal fever, sepsis

A dramatic decline in the prevalence of serious infection caused by group A streptococci has been observed throughout most of the 20th century, and *Streptococcus pyogenes* is currently an uncommon cause of maternal morbidity and mortality. However, recent reports[1–3] have suggested a resurgence of virulent group A streptococci causing sepsis, severe soft-tissue invasion, toxic shock-like syndrome, disseminated intravascular coagulation, and death. In view of the apparent reemergence of classic childbed sepsis due to this organism, we have written this review to emphasize the return of a historically important pathogen in the annals of puerperal infection.

In 1772, John Leake[4] first recognized that puerperal fever was contagious. Later that century, Alexander Gordon[5] of Aberdeen suggested that puerperal fever was a communicable disease. In 1843, Oliver Wendell Holmes[6] described the contagiousness of puerperal fever as "a momentous fact which is no longer to be considered as a subject for trivial discussion. . . ." He became convinced that childbed fever was contagious when he witnessed the death of a physician who, prior to his death, performed an autopsy on a woman with puerperal fever and attended several other parturients with similar infection. A controversy ensued and continued for many years between Holmes and American obstetricians Hugh Lennox Hodge and Charles Meigs, who vehemently opposed the theory that doctors could spread this deadly disease to their patients.

In the mid-19th century, Ignaz Semmelweis[7] noticed that puerperal fever was significantly more common in 1 of the 2 maternity divisions of the Vienna Lying-In Hospital; patients attended by medical students were more frequently afflicted with childbed fever than were patients cared for by midwives. Semmelweis[7] was therefore convinced that childbed fever could be spread from person to person and insisted that students wash their hands in chlorine solution before examining women in labor. Remarkably, maternal mortality decreased from 5% to 1.3% after implementation of this measure. Eighteen years later, Pasteur demonstrated that the disease described by Holmes[6] and Semmelweis[7] was caused by the same hemolytic streptococcus that was responsible for erysipelas, scarlet fever, and surgical wound infections.[8]

Although obstetrical texts from the beginning of the 20th century contain descriptions of what is now considered puerperal sepsis due to group A

**Address correspondence/reprint requests to Dr. Lawrence Nathan, Department of Gynecology and Obstetrics, Emory University School of Medicine, 69 Butler Street, S.E., Atlanta, GA 30303.**

Received February 8, 1994

Accepted April 13, 1994

Review Article streptococci, similar descriptions are not available in modern texts.[9–11] For example, J. Whitridge Williams,[12] in the 1st edition of *Williams Obstetrics* in 1903, described *S. pyogenes* as the most frequent cause of epidemic and fatal puerperal infection. He distinguished these infections from those caused by anaerobic bacteria by emphasizing that the latter were characterized by "putrefaction" (suppuration). Williams[12] observed that "the local changes of virulent (aerobic) streptococcal infections are comparatively slight, the process rapidly spreading through the lymphatics or veins past the uterus and giving rise to a peritonitis or a general systemic infection." He[13] continued, "In a certain number of cases the infection is so virulent that the organisms do not have a chance to become localized to any one organ, and both they and their toxins are found in abundance in the circulating blood, with very slight implication (involvement) of the uterus."

In the 1930s, Fry advanced our understanding of puerperal infection with his careful studies of the morbid anatomy in fatal infections.[14] He showed that each of the more common organisms tended to produce its own pathologic lesions. Fry's work gave rise to a better clinical understanding of these common infections, particularly of aerobic and anaerobic puerperal infections. The characteristic findings in aerobic streptococcal infections included extreme invasiveness such that the organisms rapidly spread through the uterine wall with hardly any inflammatory reaction to halt them, quickly reaching the peritoneal cavity and pelvic tissues. Such aerobic streptococcal infections were also characterized by early onset and relatively severe systemic illness, yet unobtrusive localized clinical findings. Specifically, high pyrexia and rapid pulse rate were typically most prominent, while other clinical findings, with the exception of paralytic ileus, were lacking. Fry also observed innumerable foci of bacteria within the uterine wall that he concluded escaped into the bloodstream so that a sustained septicemia resulted. In contrast, anaerobic streptococcal puerperal infections were characterized by prominent clinical findings that included putrid lochia, suppurative wounds, and pelvic abscesses. Puerperal women with anaerobic infections were typically not overwhelmed at the outset, as is seen with aerobic infections. Anaerobic infections typically progressed to abscess formation, which often included sites as distant as the lung. Pelvic thrombophlebitis along with thromboembolism was the proposed mechanism for lung involvement.

Also in the 1930s, Rebecca C. Lancefield[15] reported that streptococci could be differentiated into several groups. Lancefield's[15] group A β-hemolytic streptococcus is now recognized as the organism responsible for a variety of human diseases including puerperal sepsis and thought to be the organism responsible for the epidemics of puerperal infections in the past.

In the early 20th century, epidemics of group A streptococcal puerperal infection occurred with variable frequency and intensity. The death rate in Britain from puerperal infection was 1–2/1,000 between 1925 and 1935,[16] while an epidemic in New York's Sloan Hospital described by Watson[17] had a fatality rate of 36%. Over the subsequent 2–3 decades, aerobic streptococcal puerperal infections became a rarity that even antedated the availability of sulfa and penicillin. This decline was presumed to be related to development of knowledge on the contagiousness of aerobic streptococci resulting in the use of aseptic techniques during parturition. Moreover, part of the improvement in maternal infections was attributed to the better management of labor and elimination of those procedures that contributed to prolonged labor.[18]

In the preantibiotic era, obstetricians were concerned about gram-positive aerobes such as group A β-hemolytic streptococci (*S. pyogenes*) and anaerobes (*Peptostreptococcus*) such as *S. putridus*.[19] All these organisms shared a remarkable susceptibility to penicillin, and the introduction of penicillin into clinical practice in the mid-1940s greatly reduced clinical concerns about infections due to these pathogens.[20] Indeed, contemporary views on the pathogenesis of puerperal infections changed significantly in the postantibiotic period with group A β-hemolytic streptococci being infrequently recovered from women with postpartum metritis, and most such infections occurred only after cesarean section.[21] At our institution, most puerperal infections follow cesarean section, are typically polymicrobial (2.5 bacterial species/infection), and are associated with suppuration.[22] The most frequent bacteria isolated in these infections are *Peptostreptococcus, Bacteroides,* and *Enterobacteriaceae*. Indeed, group A β-hemolytic streptococci were isolated from only 2% of women developing puerperal infection following cesarean section, and heretofore we have not witnessed septic shock due to this organism. During the 1960s, '70s, and early '80s, puerperal infections due to group A streptococci became sporadic with only minor geographic epidemics that were limited and controlled[23–26] such that group A streptococcus was considered an infrequent cause of serious puerperal infections. For example, in 1981, Blanco and colleagues[27] reported that group A streptococcus was isolated from 3.3% of patients with puerperal endometritis.

Beginning in the mid-1980s, some investigators suggested that aerobic streptococci were reemerging as a cause of life-threatening soft-tissue infections. Stevens and co-workers[1] reported an outbreak of 20 group A β-hemolytic streptococcal infections in the Rocky Mountain region. These infections were remarkable because of the severity of the soft-tissue destruction and associated life-threatening systemic toxicity. The mortality rate was 30% despite the median age of the patients being 36 years with no underlying evidence of immune incompetence. They[1] postulated that the historical disappearance of serious streptococcal infections was partially correlated with the disappearance of type A exotoxin produced by *S. pyogenes*. The exotoxin, also called scarlet fever toxin, is believed to cause life-threatening systemic effects due to group A β-hemolytic streptococci. Other investigators[2,28–31] have also observed a resurgence of *S. pyogenes* infections with complications, including rheumatic fever, sepsis, and toxic shock-like syndrome. Cleary and co-workers[32] investigated the association of the return of scarlet fever toxin, systemic toxicity, and the possibility that a new highly virulent clone of *S. pyogenes* had emerged. Using restriction enzyme methodology and gene probes, they studied isolates from patients with sepsis and compared these with *S. pyogenes* isolates not associated with sepsis. They observed that streptococcal strains from patients with sepsis are a unique clone capable of producing exotoxin A, which is proposed to explain the apparent return of life-threatening group A β-hemolytic streptococci.

Importantly, group A streptococci have increasingly been associated with life-threatening infections on obstetric services on both sides of the Atlantic. In Europe, virulent group A β-hemolytic streptococci causing sepsis, soft-tissue infection, toxic shock syndrome, disseminated intravascular coagulation, and maternal death have been reported,[33–36] while in the United States, toxic shock due to this organism has been described in San Antonio, TX,[37] and Chapel Hill, NC.[38] Recently, Silver and associates[3] described 2 patients with life-threatening puerperal infection due to group A β-hemolytic streptococcus. Both women presented with bacteremia and shock, failed aggressive medical intervention, and required hysterectomy. Finally, we[39] reported 2 pregnancies complicated by group A β-hemolytic streptococcal sepsis after almost 2 decades without such infections at our hospital, during which time almost 200,000 women have been delivered.

It therefore appears that puerperal sepsis caused by group A β-hemolytic streptococcus, recently considered to be of historic interest only, has resumed importance and should be returned to obstetric concepts of contemporary causes of severe puerperal infection. The recent increase in both the frequency and virulence of group A streptococcal infections serves notice that this pathogen is with us today.

### REFERENCES

1. Stevens DL, Tanner MH, Winship J, et al.: Severe group A streptococcal infections with a toxic shock like syndrome and scarlet fever toxin A. N Engl J Med 321: 1–7, 1989.
2. Cone LA, Woodard DR, Schlievert PM, Tomory GS: Clinical and bacteriologic observations of a toxic shock-like syndrome due to *Streptococcus pyogenes*. N Engl J Med 317:146–193, 1987.
3. Silver RM, Heddleston LN, McGregor JA, Gibbs RS: Life-threatening puerperal infection due to group A streptococci. Obstet Gynecol 79:894–896, 1992.
4. Leake J: Practical Observations on Child-Bed Fever; Also on the Nature and Treatment of Uterine Haemorrhages, Convulsions, and Such Other Acute Diseases as Are Most Fatal to Women During the State of Pregnancy. London: J Walter, 1772.
5. Gordon A: A Treatise on the Epidemic Puerperal Fever of Aberdeen. London: CG & J Robinson, 1795.
6. Holmes OW: The contagiousness of puerperal fever. N Engl J Med Surg 1:503–530, 1843.
7. Semmelweis IP: Childbed fever. (Excerpts from: The etiology, the concept and the prophylaxis of childbed fever, 1861.) Rev Infect Dis 3:808–811, 1981.
8. Watson BP: Puerperal infection. Am J Obstet Gynecol 40:584–588, 1940.
9. Cunningham FG, MacDonald PC, Gant NF, Leveno KJ, Gilstrap LC (eds): Williams Obstetrics. 19th Ed. Norwalk, CT: Appleton & Lange, 1993.
10. Ledger WJ (ed): Infection in the Female. 2nd Ed. Philadelphia: Lea & Febiger, 1986.

11. Gilstsrap LC III, Faro S (eds): Infections in Pregnancy. New York: Alan R. Liss, Inc., 1990.
12. Williams JW (ed): Pathology of the puerperium. In: Williams Obstetrics. 1st Ed. New York: D. Appleton & Co., p 764, 1903.
13. Williams JW (ed): Pathology of the puerperium. In: Williams Obstetrics. 1st Ed. New York: D. Appleton & Co. p 780, 1903.
14. Gibberd GF: Puerperal sepsis, 1930–1965. J Obstet Gynaecol Br Commonw 73:1–10, 1966.
15. Lancefield RC: A serological differentiation of human and other groups of hemolytic streptococci. J Exp Med 57:571–595, 1933.
16. Rubenstein A: Subtle poison: The puerperal fever controversy in Victorian Britain. Hist Stud 20:420–438, 1983.
17. Watson BP: An outbreak of puerperal sepsis in New York City. Am J Obstet Gynecol 16:157–179, 1928.
18. Douglas RG, Davis IF: Puerperal infection. Etiologic, prophylactic and therapeutic considerations. Am J Obstet Gynecol 51:352–371, 1946.
19. Schwarz OH, Dieckmann WJ: Puerperal infection due to anaerobic streptococci. Am J Obstet Gynecol 13:467–485, 1977.
20. Ledger WJ: A historical review of pelvic infections. Am J Obstet Gynecol 158:687–693, 1988.
21. Ledger WJ: Hospital-acquired obstetric infections. In Zuspan FP (ed): Infection in the Female. 1st Ed. Philadelphia: Lea & Febiger, p 206, 1977.
22. Gilstrap LC III, Cunningham FG: The bacterial pathogenesis of infection following cesarean section. Obstet Gynecol 53:545–549, 1979.
23. Jewett JF, Rerd DE, Safon LE, Easterday CL: Childbed fever—A continuing entity. JAMA 206:344–350, 1968.
24. Ledger WJ, Headington JT: Group A beta hemolytic streptococcus. An important cause of serious infections in obstetrics and gynecology. Obstet Gynecol 39:474–482, 1972.
25. Ogden E, Amstey MS: Puerperal infection due to group A beta hemolytic streptococcus. Obstet Gynecol 52:53–55, 1978.
26. McGregor J, Ott A, Villard M: An epidemic of "childbed fever." Am J Obstet Gynecol 150:385–388, 1984.
27. Blanco JD, Gibbs RS, Castaneda YS: Bacteremia in obstetrics: Clinical course. Obstet Gynecol 56:621–625, 1981.
28. Bartter T, Dascal A, Carroll K, Curley FJ: "Toxic strep syndrome": A manifestation of group A streptococcal infection. Arch Intern Med 148:1421–1424, 1988.
29. Bisno AL: Group A streptococcal infections and acute rheumatic fever. N Engl J Med 325:783–793, 1991.
30. Veasy LG, Wiedmeier SE, Orsmond GS, et al.: Resurgence of acute rheumatic fever in the intermountain area of the United States. N Engl J Med 316:421–427, 1987.
31. Stollerman GM: Changing group A streptococci. The reappearance of streptococcal "toxic shock." Arch Intern Med 148:1268–1270, 1988.
32. Cleary PP, Kaplan EL, Handley JP, Wlazlo A, Kim MH, Hauser AR, Schlievert PM: Clonal basis for resurgence of serious *Streptococcus pyogenes* disease in the 1980s. Lancet 339:518–521, 1992.
33. Martens PR, Mullie A, Goessens L: A near-fetal case of puerperal sepsis. Anaesth Intensive Care 19:108–110, 1991.
34. Acharya U, Lamont CAR, Cooper K: Group A beta-haemolytic streptococcus causing disseminated intravascular coagulation and maternal death. Lancet 1:595, 1988.
35. Kavi J, Wise R: Group A beta-haemolytic streptococcus causing disseminated intravascular coagulation and maternal death. Lancet 1:993–994, 1988.
36. Swingler GR, Bigrigg MA, Hewitt BG, McNulty CAM: Disseminated intravascular coagulation associated with group A streptococcal infection in pregnancy. Lancet 1:1456–1457, 1988.
37. Whitted RW, Yeomans ER, Hankins GDV: Group A β-hemolytic streptococcus as a cause of toxic shock syndrome. J Reprod Med 35:558–560, 1990.
38. Dotters DJ, Katz VL: Streptococcal toxic shock associated with septic abortion. Obstet Gynecol 78:549–550, 1991.
39. Nathan L, Peters MT, Ahmed AM, Leveno KJ: The return of life-threatening puerperal sepsis caused by group A streptococci. Am J Obstet Gynecol 169:571–572, 1993.
